# The kinetics of gut microbial community composition in patients with irritable bowel syndrome following fecal microbiota transplantation

**DOI:** 10.1371/journal.pone.0194904

**Published:** 2018-11-14

**Authors:** Tarek Mazzawi, Gülen Arslan Lied, Dag André Sangnes, Magdy El-Salhy, Johannes R. Hov, Odd Helge Gilja, Jan Gunnar Hatlebakk, Trygve Hausken

**Affiliations:** 1 Section of Gastroenterology, Department of Medicine, Haukeland University Hospital, Bergen, Norway; 2 Norwegian Competence Centre for Functional Gastro-Intestinal Disorders, Section of Gastroenterology, Department of Medicine, Haukeland University Hospital, Bergen, Norway; 3 Center for Nutrition, Department of Clinical Medicine, University of Bergen, Bergen, Norway; 4 Section of Gastroenterology, Department of Medicine, Stord Hospital, Helse-Fonna, Stord, Norway; 5 Norwegian PSC Research Center, Section of Gastroenterology and Research Institute of Internal Medicine, Division of Surgery, Inflammatory Diseases and Transplantation, Oslo University Hospital and University of Oslo, Oslo, Norway; University Hospital Llandough, UNITED KINGDOM

## Abstract

**Background:**

Gut microbiota alterations are important in irritable bowel syndrome (IBS). The aim was to investigate the effect of fecal microbiota transplantation (FMT) on gut microbiota and the symptoms in patients with IBS.

**Material and methods:**

The study included 13 IBS patients according to Rome III criteria and 13 healthy donors. Freshly donated feces were administered to the descending part of the duodenum via a gastroscope. Feces were collected from donors and patients before FMT, and from the patients at 1, 3 and 12 weeks and donors and patients at 20/28 weeks after FMT. Microbiota analysis was performed using GA-map Dysbiosis test (Genetic Analysis AS, Oslo, Norway). The patients completed the following questionnaires before and at the aforementioned weeks after FMT: IBS Symptom Questionnaire (IBS-SQ), IBS-Symptom Severity Scoring system (IBS-SSS), Short Form of Nepean Dyspepsia Index (SF-NDI), Bristol stool form scale, the Eysenck Personality Questionnaire-Neuroticism and Hospital Anxiety and Depression.

**Results:**

Donors and IBS patients had significantly different bacterial strain signals before FMT (*Ruminococcus gnavus*, *Actinobacteria* and *Bifidobacteria*) that became non-significant after 3 weeks following FMT. The changes in gut microbiota were similar between donors and patients at 20/28 weeks after FMT. Thus, patients’ microbiota profiles became more-or-less similar to donors.

The scores of all the questionnaires were significantly improved at all time points following FMT. No reported adverse effects.

**Conclusions:**

FMT was associated with a change in gut microbiota and improvement in IBS symptoms and quality of life lasting for up to 28 weeks.

**Trial registration:**

**ClinicalTrials.gov ID:** NCT03333291

## Introduction

Irritable bowel syndrome (IBS) is a common chronic gastrointestinal (GI) disease, affecting 10–20% of the adult population leading to significant morbidity and huge costs for the society [1]. The pathogenesis of IBS is unclear, but it is believed to be multifactorial; and includes altered gut microbiota, [2, 3] abnormal enteroendocrine cells of the GI tract [4], mucosal low-grade inflammation, [5, 6] genetic predisposition [7] and diet [8, 9]. Some reports describe that postinfectious IBS (PI-IBS) occurs in 10–30% of patients following acute gastroenteritis, suggesting that alterations in the gut microbiota may play a role in the pathogenesis of this type of IBS [10–12].

Gut microbiota play an important role in maintaining health, regulating cellular immunity and energy metabolism [10]. Recent studies have shown that the gut microbiota are involved in GI and non-GI disorders (e.g. obesity, atherosclerosis and type II diabetes mellitus) [13–15]. The important role of alterations in the gut microbiota in IBS [2, 3] has led to increased interest in probiotic [16] and antibiotic [17] treatment approaches.

Fecal microbiota transplantation (FMT), the infusion of a fecal preparation from a healthy donor into the GI tract of a human recipient may alter the gut microbiome (the bacterial gene content) of the new host by re-establishing the balance in the gut microbiota [10]. It is speculated that human feces from a healthy donor may constitute “the ultimate human probiotic” [10], thus proposing FMT as a treatment option for conditions where an altered gut microbiota has been detected, including IBS [10, 18, 19]. FMT was first reported to be used for treatment of pseudomembranous colitis caused by *Micrococcus pyogenes (Staphylococcus)* in 1958 [20] and then in 1983 for *Clostridium difficile* infection [21]. Currently, FMT is widely accepted as the recommended treatment for recurrent *Clostridium difficile* enterocolitis [22]. Two new studies have shown that FMT improves the symptoms of recipient patients with IBS [23, 24] and one case report shows that the stool microbiome of the recipient resembled that of the donor following FMT [25]. Other reports about the use of FMT in selective cases of ulcerative colitis [26, 27], chronic fatigue syndrome [10] and autism [28] have resulted in positive outcomes [10].

The aims of the current study were to investigate the effect of FMT on i) the characteristics and kinetics of the gut microbiota in IBS patients, and ii) the symptoms and quality of life in IBS patients.

## Material and methods

### Eligible patients

A recipient group (*n* = 16) included both male and female patients, aged between 18–70 years, who met Rome III criteria for the diagnosis of IBS with moderate to severe abdominal symptoms as defined by IBS-Symptom Severity Scoring system (IBS-SSS) score *>*175 [29] and were referred to the gastroenterology outpatient clinic, Haukeland University Hospital, Bergen, Norway. The exclusion criteria included history of inflammatory bowel diseases, GI malignancy, blood in stool, an immunocompromised state, a history of opportunistic infections within 1 year prior to FMT, oral thrush, or disseminated lymphadenopathy. Patients who were scheduled for abdominal surgery, pregnant or lactating women and patients taking probiotics or antibiotics within 4 weeks prior to fecal installation were also excluded.

### Donors

A donor group of healthy family members, males and females who were over 18 years of age was included. The exclusion criteria of the donors were pregnancy, history of inflammatory bowel diseases, IBS, chronic abdominal pain, GI malignancy, diarrhea, blood in stool, antibiotic and probiotic use within 4 weeks prior to FMT, an immunocompromised state, history of opportunistic infections within 1 year prior to FMT, oral thrush and disseminated lymphadenopathy.

The study was performed in accordance with the Declaration of Helsinki [30] and was approved by the Regional Committee for Medical and Health Research Ethics in Western Norway (reference no.: 2013/1497). All participants provided written informed consent. According to the Norwegian legislation, clinical trials concerning fecal transplantation are not regarded as drug clinical trial. When the study started we were unfortunately not aware of the requirements of registering non-drug clinical trials. Hence this trial was registered retrospectively at ClinicalTrials.gov (ID: NCT03333291).

### Study design

The FMT procedure was done only once and fecal samples were analyzed across several time points before FMT at screening (week -1) and FMT day (week 0), and then after FMT at weeks 1, 3, 12 and 28 weeks. The scheduled study visits are outlined in Table 1. The donors and patients completed several questionnaires and delivered fresh stool samples soon (a couple of hours) after defecation at screening and during visits after FMT as outlined in Table 1. The patients received special containers to preserve their stool in and were informed to place them in the refrigerator (4°C) if it will take longer than a couple of hours before delivery. The patients were informed not to apply any changes to their diet or life style and to report any bout of new infections and/or use of new medications during the study.

**Table 1:** The intervention plan and timing of the visits.

Participants	Screening (-1 week)	FMT-day (week 0)	Visit 1 (week 1)	Visit 2 (week 3)	Visit 3 (week 12)	Visit 4 (week 20/28)
**Patients**						
	IBS-SSS	IBS-SSS	IBS-SSS	IBS-SSS	IBS-SSS	IBS-SSS
	IBS-SQ		IBS-SQ	IBS-SQ		
	History and physical examination	Bristol stool form scale	Bristol stool form scale	Bristol stool form scale	Bristol stool form scale	Bristol stool form scale
	Blood tests	SF-NDI		SF-NDI		SF-NDI
	Stool tests	EPQ-N-12		EPQ-N-12		EPQ-N-12
		HAD		HAD		HAD
		Fresh stool for storage (-80°C) and analysis	Fresh stool for storage (-80°C) and analysis	Fresh stool for storage (-80°C) and analysis	Fresh stool for storage (-80°C) and analysis	Fresh stool for storage (-80°C) and analysis
		Gastroscopy for installation of freshly donated feces				
**Donors**						
	History and physical examination	Fresh stool for donation				Fresh stool for storage (-80°C) and analysis
	IBS-SSS					
	IBS-SQ					
	Blood tests					
	Stool tests					

IBS-SSS: Irritable bowel syndrome-symptom severity scale; IBS-SQ: IBS symptom questionnaire; SF-NDI: short form-Nepean dyspepsia index; EPQ-N-12: The Eysenck Personality Questionnaire-Neuroticism; HAD: hospital anxiety and depression.

### Screening

Screening of the donors and the patients was scheduled one week before FMT. All of the donors and patients filled out symptom questionnaires (Table 1), received physical examinations and were screened (in blood and stool) for previous exposure to contagious infectious agents, inflammation and other organic diseases. Screening of the donors’ blood included serologic testing for hepatitis A, B, C, human immunodeficiency virus (HIV), Epstein-Barr virus and cytomegalovirus. The blood from the patients was tested and included: Hemoglobin, leucocytes, platelets, creatinine, aspartate transaminase (AST), alanine transaminase (ALT), International Normalized Ratio (INR), electrolytes and chromogranin A. Stool samples from the donors and patients were examined for fecal calprotectin, cultured for enteric bacterial pathogens and screened for viruses and parasites.

### The FMT procedure

On FMT day, the patients brought >60 g of fresh feces from their donors along with 60 g of their own feces before transplantation. Only 30 g of donor feces were used to prepare the fecal suspension by mixing them with 60 ml of normal saline. The remaining donor feces and feces from the patients were stored at -80°C until they were analyzed for microbial analysis. The patients completed several questionnaires before FMT (Table 1). Gastroscopy was performed on the patients (after an overnight fast) to install 60 ml of fecal suspension followed by 60 ml of normal saline in the descending part of the duodenum distal to the papilla Vateri. All of the gastroscopies were performed by an endoscopist (T.M., G.A.L. or T.H.) at the gastrolab, Haukeland University Hospital, Bergen, Norway. The second visit was planned at week 3, instead of week 4 as outlined in the original protocol, due to practical reasons, and only 30 g of donor feces was used only once, as outlined in the original protocol (S1 File), in accordance with previous recommendations [10, 31].

### Gut microbiota analysis

Gut microbiota analysis was performed using the GA-map Dysbiosis test (Genetic Analysis AS, Oslo, Norway) by algorithmically assessing fecal bacterial abundance and profile (dysbiosis index, DI), and potential deviation in the microbiome from normobiosis. [32] Briefly, GA-map Dysbiosis test is based on fecal homogenization, mechanical bacterial cell disruption and automated total bacterial genomic DNA extraction using magnetic beads. DI is based on 54 DNA probes targeting more than 300 bacterial strains based on their 16S rRNA sequence in seven variable regions (V3–V9). Twenty-six bacteria probes are species specific, 19 detect bacteria on genus level, and 9 probes detect bacteria at higher taxonomic levels. Probe labeling is by single nucleotide extension and hybridization to complementary probes coupled to magnetic beads, and signal detection by using BioCode 1000A 128-Plex Analyzer (Applied BioCode, Santa Fe Springs, CA, USA). A DI above 2 shows a microbiota profile that differs from that of the normobiotic reference collection (DI 1–2: non-dysbiosis, DI 3: moderate, DI 4–5: severe dysbiosis) [32].

### Questionnaires

Gastrointestinal symptoms and bowel habits were evaluated using IBS-SSS [29] in which a decrease of 50 points in IBS-SSS score using a visual assessment scale (VAS) from baseline (before FMT) correlated with improvement in clinical symptoms, and IBS symptom questionnaire (IBS-SQ) [33, 34] that was completed on the day of screening and then daily for 20 days after FMT. Responders and late-responders were patients who achieved a reduction of >50 points in IBS-SSS score after 1 and 3 weeks following FMT, respectively [29]. Non-responders were those who achieved <50 points in IBS-SSS score following FMT at any time period compared to baseline.

Stool consistency was evaluated using Bristol stool form scale [35], which ranges from 1 (constipation) to 7 (diarrhea). Quality of life (QoL) was assessed using Short Form of Nepean Dyspepsia Index (SF-NDI) questionnaire [36]. Psychometric evaluation was performed using the Eysenck Personality Questionnaire-Neuroticism (EPQ-N-12) with a cut-off value of 4 [37], and Hospital Anxiety and Depression (HAD) where scores >8 in either subscale were considered to indicate anxiety or depression, respectively [38, 39].

### Statistical analysis

Graphpad Prism 6 (GraphPad Software Inc., La Jolla, CA, USA) was used for all analysis. Kruskal-Wallis non-parametric test with Dunn’s post test and Mann-Whitney U test were used to analyse the data between the donors and patients before and after FMT. One-way ANOVA with repeated measures and Paired *t*-test was used to analyze the data for the patients before FMT and each visit after FMT. Multiple *t*-test corrected using Holm-Sidak method, was used to compare between the bacterial signals of responders and non-responders. *P <* 0.05 is considered to indicate a statistically significant difference. Cluster analysis and principal component analyses (PCA) were used to visualize the microbiota data, showing the extent to which microbial communities share branch length.

## Results

### Participants

**Fig 1:**
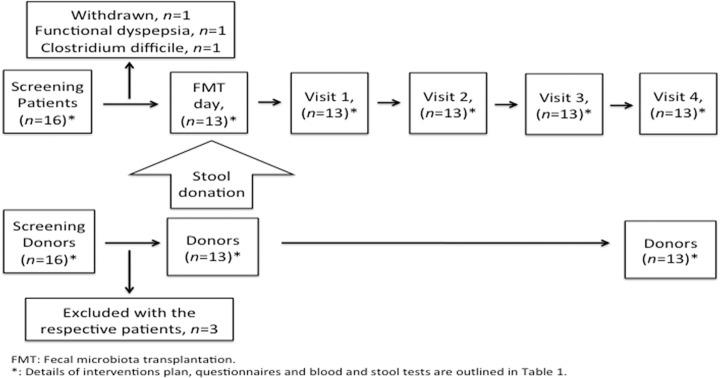
Study flow chart.

The recipients group included 16 patients with IBS and the donors group included 16 healthy subjects. Participants of both groups were recruited after fulfilling the inclusion’s criteria and, most importantly, none has used antibiotics during the past 6 months prior to inclusion in the study (Fig 1). Three, originally recruited, patients were excluded after withdrawing their consent to participate for practical reasons (*n* = 1), being diagnosed with functional dyspepsia (*n* = 1) and finding *Clostridium difficile* in stool culture (*n* = 1). Hence, 13 patients (9 males and 4 females, mean age of 32 years and age range of 20–44 years) and 13 donors (6 males and 7 females, mean age of 33 years and age range of 20–42 years) completed the whole study, and filled out the questionnaires and delivered fecal samples as previously explained. All of the patients had IBS mostly diarrhea-predominant, in which six patients had PI-IBS (after a local Giardia outbreak in Bergen in 2004 [40]) and seven patients had idiopathic IBS. The last visit was originally scheduled at 28 weeks after FMT, but 4 patients and also their respective donors were scheduled for a last visit at 20 weeks instead of 28 weeks after FMT due to practical reasons. The blood tests and stool cultures of both donors and patients were normal prior FMT and control blood tests for the patients were also normal at the end of the study. Detailed health and symptom questionnaires were provided to both groups at screening day and only to the patients following FMT throughout the study. We asked both the patients and donors to report any changes in their diet, life style, medications or health history during the whole study. Neither group reported any such changes during study participation.

### Gut microbiota

**Fig 2:**
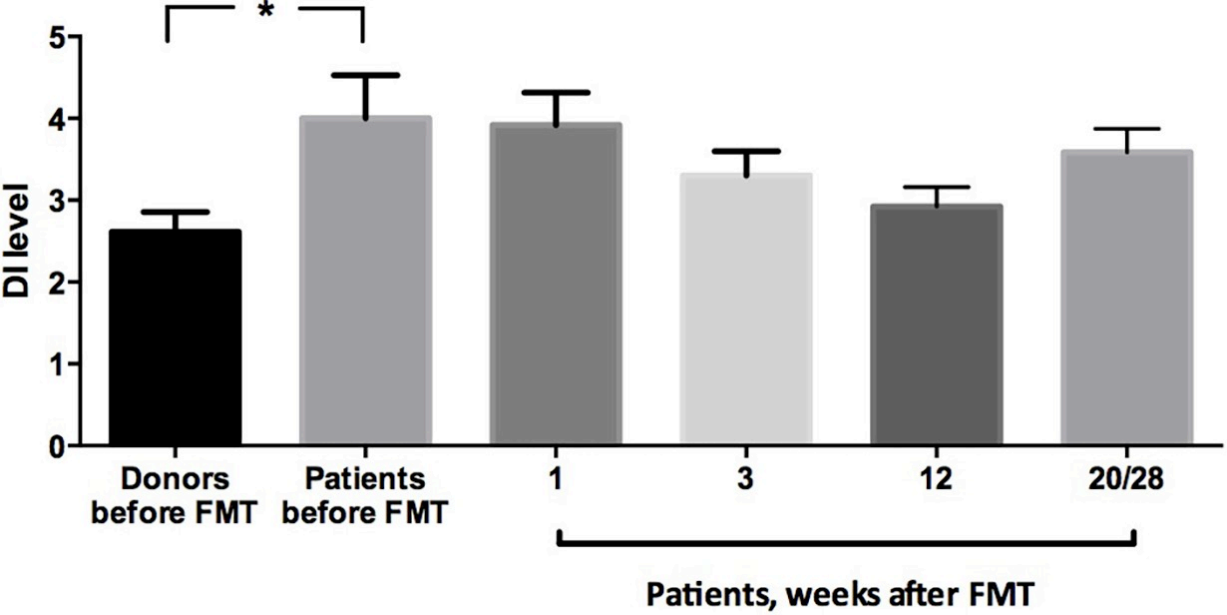
Dysbiosis index of the donors before fecal microbiota transplantation (FMT) and for the patients before FMT (day 0) and following FMT (weeks 1, 3, 12 and 20/28).

At baseline, the patients had significantly higher DI than the donors (4±0.5 and 2.6±0.2, respectively, *P* = 0.046, Fig 1). Following FMT, the DI for the patients gradually decreased to 3.9±0.4 at week 1, then 3.3±0.3 at week 3 and 2.9±0.2 at week 12 but then increased again to 3.5±0.3 at week 20/28. The changes in the DI comparing between the patients following FMT and the donors were not statistically significant (Fig 2).

**Fig 3:**
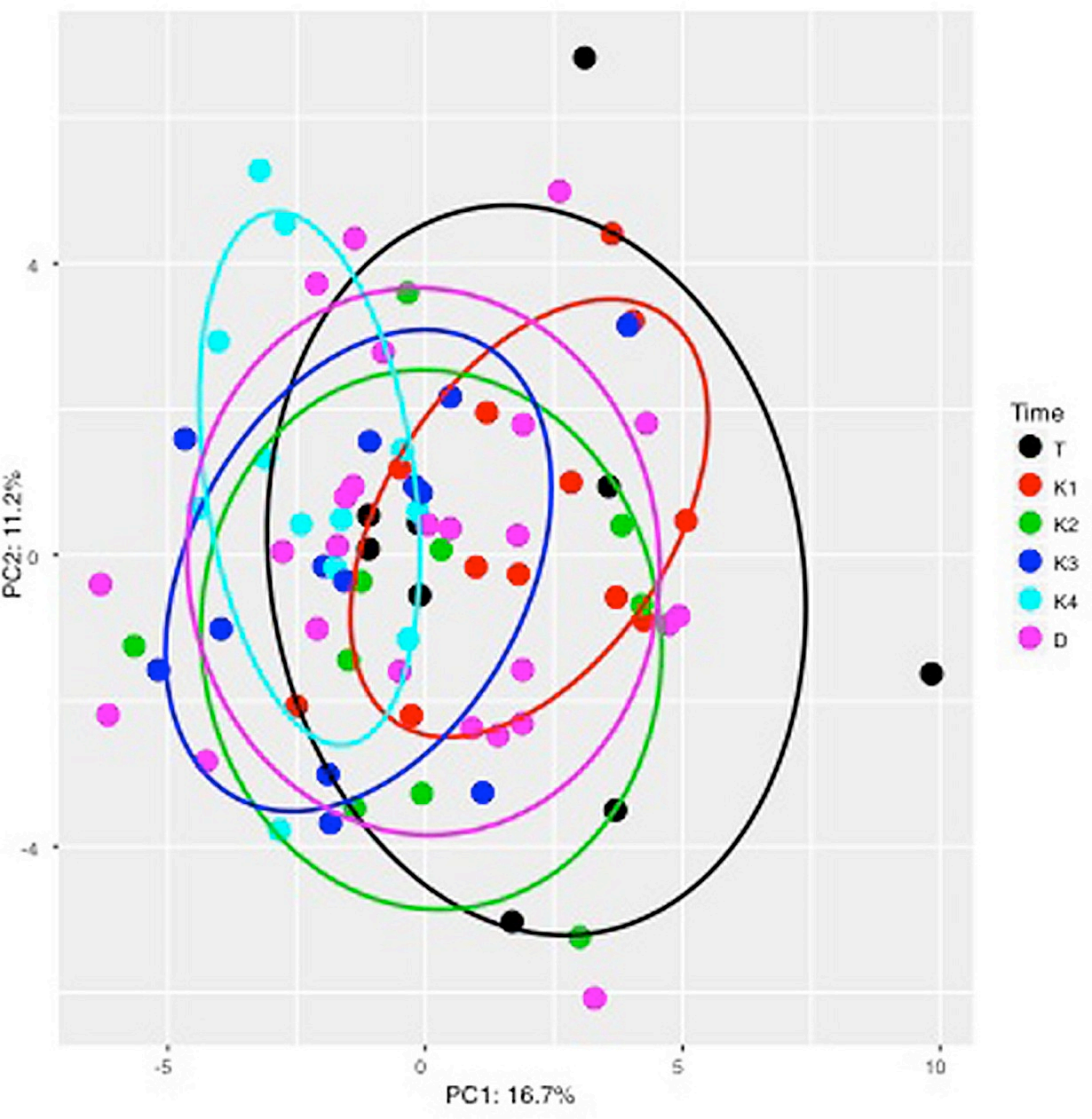
Scores for the first two principal component analysis (PCA) of fecal microbiota in donors at the beginning of the study and patients with irritable bowel syndrome after fecal microbiota transplantation (FMT, *n* = 13). Data have been centered within each donor series to remove donor differences for this analysis. Data are shown as donor (D: pink dots), FMT day (T: black dots), visit at week 1 (K1: red dots), visit at week 3 (K2: green dots), visit at week 12 (K3: dark blue dots) and visit at week 20/28 (K4: light blue dots). Each dot represents data from one patient for one visit. The first two PCs account for 27.9% (sum of PC1 and PC2) of the variation. The colored ellipses demonstrate the 68% confidence interval for PC1 and PC2. The PCA scores show that FMT seem to have an effect on the gut microbiota as systematic change is going forward.

Donors and IBS patients had significantly different bacterial signals before FMT, namely, signals for *Ruminococcus gnavus*, *Actinobacteria* and *Bifidobacteria*, which became non-significantly different after 3 weeks following FMT (Table 2). At weeks 12 and 20/28, new bacterial strains in the patients feces; namely *Bacteroides/Prevotella*, *Alistipes*, *Actinobacteria* and *Bifidobacteria* became significantly different from that of the donor at the beginning of the study, as shown in Table 2, but not statistically different from that of the donors at the end of the study at week 20/28 (*P* = 0.09, 0.08, 0.6, 0.14 and 0.9, respectively). The signal levels of *Actinobacteria* and *Bifidobacteria* increased significantly towards the levels measured for the donors and lasted for 12 weeks after FMT but then significantly decreased at week 20/28. The PCA scores plot of the gut microbiota profiles corrected for sample differences showed a gradual shift of the gut microbiota profile over time (Fig 3).

**Table 2:** Characteristics of the fecal bacterial signals between the donors at the beginning and end of the study, and patients in weeks 0, 1, 3, 12 and 20/28. The left part of the table shows the bacterial signals given for donors and recipients at different time points before and after FMT and the directionality towards or away from that of the donors at baseline. The right part of the table shows the *P*-values when comparing the bacterial signals for the recipients at different time points to that for the donors at baseline.

Bacteria strain	Donors, Beginning of study, *n* = 13	Patients					Donors, End of study, *n* = 10	P^a^ Before FMT	P^b^ After 1 week	P^c^ After 3 weeks	P^d^ After 12 weeks	P^e^ After 20/28 weeks	P^f^ Donors
		FMT day, *n* = 9	After 1 week, *n* = 12	After 3 weeks, *n* = 9	After 12 weeks, *n* = 13	After 20/28 weeks, *n* = 12							
***Ruminococcus gnavus***	4.6±1.1	40±15.6	8±2.3	15.3±9	25±17	8.1±1.8	8.8±2	**0.015**	>0.9	0.15	0.44	>0.9	0.19
***Bacteroides***	27.2±4.1	38.9±8.3	47.1±16.6	30.9±3.7	55.4±12.7	49.3±11	42±5.2	>0.9	>0.9	>0.9	**0.02**	0.1	0.097
***Bacteroides/Prevotella***	483±51.4	634±28.5	599±25.8	551±63.9	783±49.7	731±52	788±58	0.7	>0.9	>0.9	**0.005**	**0.02**	**0.009**
***Alistipes***	100.5±16.8	100.5±23	119±17.4	140±26.8	186±11.9	208±9.6	188±15.5	>0.9	>0.9	0.9	**0.011**	**0.0006**	**0.03**
***Parabacteroides***	7.6±1.8	7.9±2.9	8.3±2	14.6±5.4	11.8±1.8	15.7±3.5	19.5±3.8	>0.9	>0.9	>0.9	0.4	0.3	**0.03**
***Actinobacteria***	287±45	66.6±13	95±23	197±54	138±29	92±23	204±57	**0.0010**	**0.007**	0.7	0.2	**0.003**	**0.018**
***Bifidobacteria***	324±57	65±13	97±25	205±57	150±34	92.5±25	241±72	**0.0011**	**0.008**	0.6	0.2	**0.004**	**0.017**
***Proteobacteria***	27±7.8	195±121.6	565±162	63.8±28.5	105±70	56.5±40	17±1.9	>0.9	**0.03**	>0.9	0.9	>0.9	0.5
***Shigella/Escherichia***	62±23	260±124	578±128	116±45	188±77	90.9±65	41±14	>0.9	**0.002**	0.8	>0.9	>0.9	0.8

Data are presented as the mean±SEM. Comparison: Kruskal-Wallis multiple comparisons test with Dunn’s post test

^**a**^ Donors at the beginning of the study vs. patients on FMT day before fecal installation.

^**b**^ Donors at the beginning of the study vs. patients 1 week after FMT.

^**c**^ Donors at the beginning of the study vs. patients 3 weeks after FMT.

^**d**^ Donors at the beginning of the study vs. patients 12 weeks after FMT.

^**e**^ Donors at the beginning of the study vs. patients 20/28 weeks after FMT.

Paired *t* test

^**f**^ Donors at the beginning vs. end of the study.

FMT: fecal microbiota transplantation.

**Fig 4:**
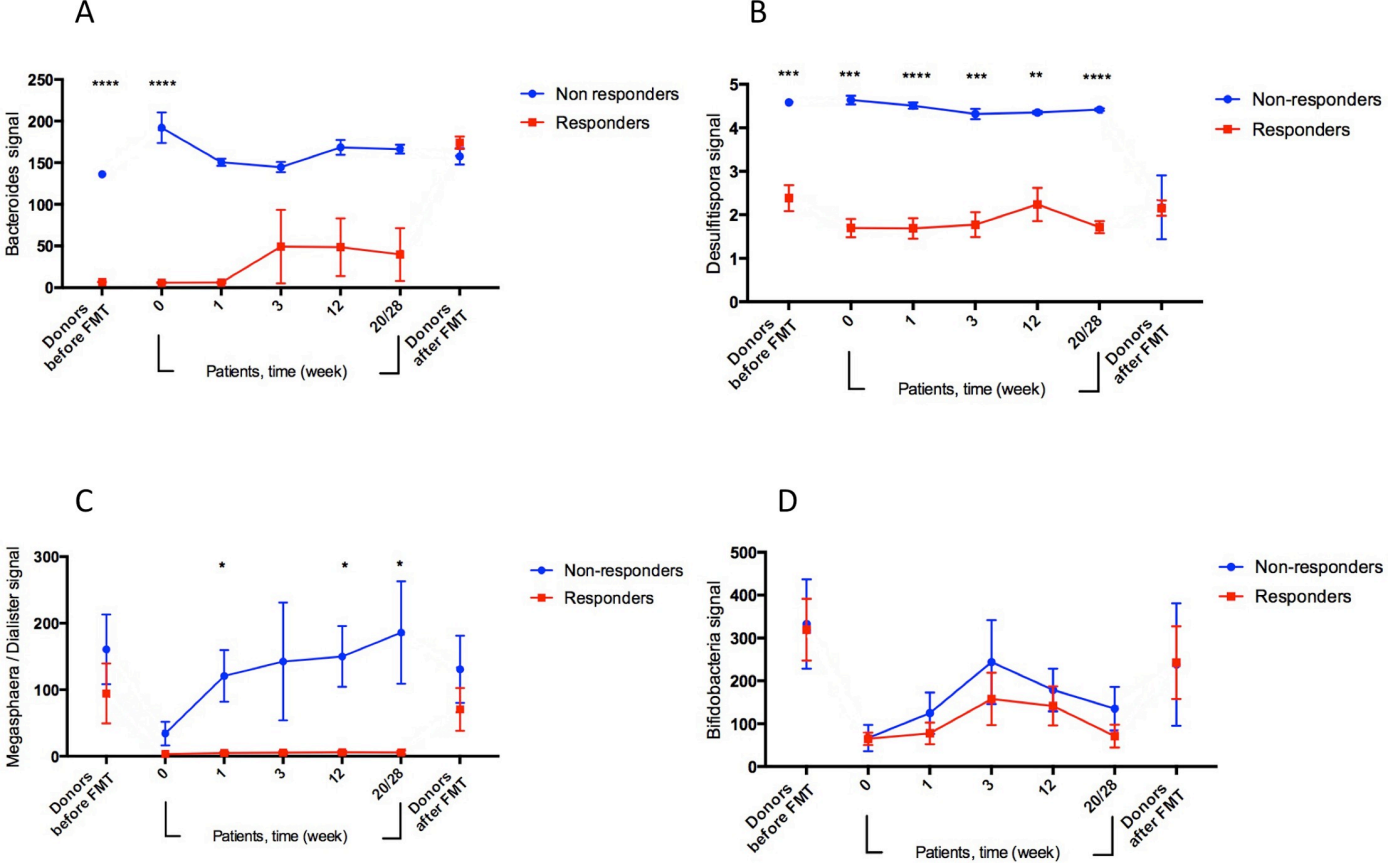
The characteristic differences of gut microbiota between responders and non-responders groups before and after FMT. The bacterial signals of (A) *Bacteroides*, (B) *Desulfitispora*, (C) *Megasphaera/Dialister* and (D) *Bifidobacteria*, in the responders and non-responders groups between the donors at the beginning of the study (before FMT), and between the patients in weeks 0, 1, 3, 12 and 20/28.

Comparing the microbiota between the groups, responders (*n* = 8) and non-responders (*n* = 5), which were defined based on achieving >50 or <50 IBS-SSS points, respectively, at week 20/28 compared to baseline, showed significant differences (adjusted *P*-values) in the *Bacteroides* signals between the donors at the beginning and the end of the study (*P*<0.0001 and 0.23, respectively) and between the patients at weeks 0, 1, 3, 12 and 20/28 (*P*<0.0001, <0.0001, 0.23, 0.08 and 0.08, respectively), Fig 4A. The respective values for *Desulfitispora* signals between the donors at the beginning and the end of the study (*P* = 0.0005 and 0.98, respectively) and between the patients at weeks 0, 1, 3, 12 and 20/28 (*P* = 0.0002, <0.0001, 0.0005, 0.0025 and <0.0001, respectively), Fig 4B, and for *Megasphaera/Dialister* signals between the donors at the beginning and the end of the study (*P* = 0.54 and 0.53, respectively) and between the patients at weeks 0, 1, 3, 12 and 20/28 (*P* = 0.13, 0.029, 0.24, 0.013 and 0.029, respectively), Fig 4C. As for *Bifidobacteria*, differences in the bacterial signals between responders and non-responders were noted, however, they were statistically not significant, Fig 4D.

### Questionnaires

**Fig 5:**
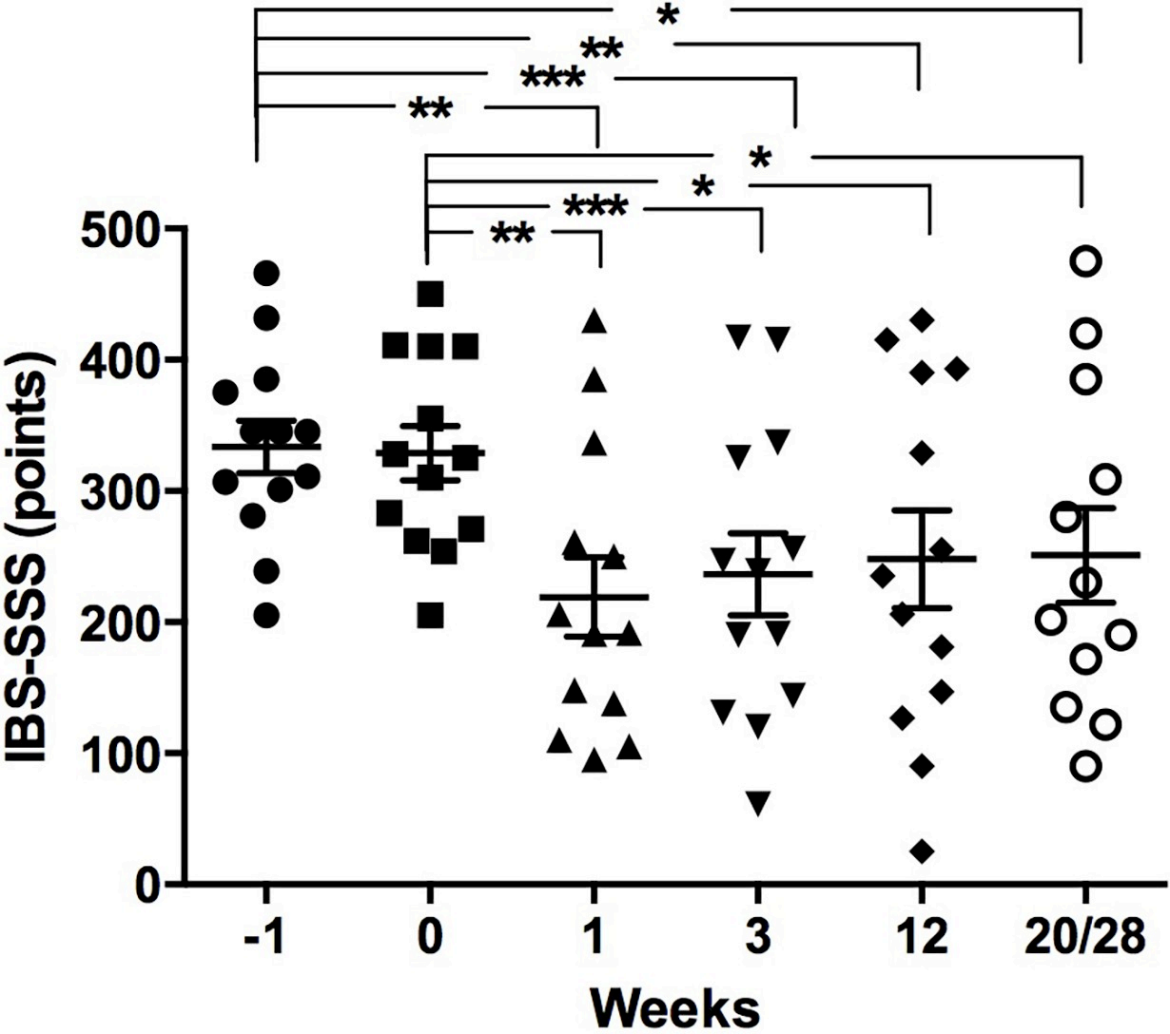
IBS-SSS scores of the patients one week before fecal microbiota transplantation (FMT, week -1), at FMT day (0), and following FMT (weeks 1, 3, 12 and 20/28).

The score of IBS-SSS (mean±SEM) for the donors was 18±8.9 and for IBS-SQ is 0.7±0.3, which indicated asymptomatic status. The score of IBS-SSS of 11 patients were considered severe (IBS-SSS score >300) and only two patients had moderate severity (IBS-SSS score = 176–300) before FMT. The score of IBS-SSS (mean±SEM) for the patients at screening day was 333.6±20. IBS-SSS scores at FMT-day and at weeks 1, 3, 12 and 20/28 after FMT and comparisons between FMT-day and at each week are presented in Table 3. Using paired *t* test showed no significant difference in the aforementioned IBS-SSS scores for the patients between screening and FMT-day (*P* = 0.45), however, a significant reduction was noted in IBS-SSS scores of the patients between screening and weeks 1, 3, 12 and 20/28 (*P* = 0.003, 0.0004, 0.0095 and 0.012, respectively). No significant differences were observed by comparing the scores in weeks 1, 3, 12 and 20/28 interchangeably between each other, Fig 5. Four out of the 13 patients did not achieve >50 points reduction in IBS-SSS scores from baseline at week 1. However, two out of these four patients were late responders and achieved >50 points reduction in IBS-SSS scores from baseline at week 3. Therefore, a total of 9, 11, 9 and 8 out of the 13 patients achieved >50 points reduction in IBS-SSS scores from baseline at weeks 1, 3, 12 and 20/28 following FMT, respectively, and were considered as responders.

**Table 3:** Scores of the patients’ questionnaires before and after fecal microbiota transplantation.

Questionnaire	FMT-day (week 0)	Week 1	Week 3	Week 12	Week 20/28)	P^a^	P^b^	P^c^	P^d^	P^e^
**IBS-SSS**	328.8±20.7	219.1±30.2	236.4±31.1	247.9±37.1	250.8±35.9	**0.008**	**0.002**	**0.0003**	**0.014**	**0.015**
**Bristol stool form scale**	4.8±0.5	3.7±0.4	3.9±0.4	4.2±0.3	3.8±0.5	**0.07**	**0.042**	**0.0075**	0.17	0.16
**SF-NDI**	34.9±2.0	-	27.5±2.3	-	28.6±2.4	**0.0045**	-	**0.0039**	-	**0.0068**
**EPQ-N-12**	4.8±0.8	-	3.7±0.8	-	4.7±1.3	0.36	-	0.07	-	0.6
**HAD, anxiety**	7.2±1.1	-	5.3±1.0	-	5.9±1.5	0.18	-	**0.016**	-	0.24
**HAD, depression**	5.3±0.98	-	3.5±0.8	-	5.3±1.2	0.08	-	**0.038**	-	>0.9

Data are presented as the mean±SEM. Comparison

^**a**^ one-way ANOVA with repeated measures; and Paired *t* test

^**b**^ FMT day (week 0) vs. week 1

^**c**^ FMT day (week 0) vs. week 3

^**d**^ FMT day (week 0) vs. week 12

^**e**^ FMT day (week 0) vs. week 20/28.

FMT: Fecal microbiota transplantation.

IBS-SSS: Irritable bowel syndrome-symptom severity scale.

SF-NDI: short form-Nepean dyspepsia index.

EPQ-N-12: The Eysenck Personality Questionnaire-Neuroticism.

HAD: Hospital Anxiety and Depression.

The scores for the following questionnaires are presented in Table 3: Bristol stool form scale, total SF‐NDI scores, EPQ-N-12 and HAD. Bristol stool form scale showed significant changes in the stool form from diarrhea type before FMT to normal following FMT (weeks 1 and 3, *P* = 0.07 and 0.04, respectively), however, no significant differences were noted between weeks 1, 3, 12 and 20/28 when comparing them interchangeably with each other. The total SF‐NDI scores showed a significant improvement in the QoL following FMT that lasted to the end of the study and no significant difference was found between weeks 3 and 20/28. The scores for EPQ-N-12 and HAD showed only significant improvement in HAD scores (anxiety and depression) 3 weeks after FMT (*P* = 0.016 and 0.038, respectively). The scores for the different domains of IBS-SQ during the first 3 weeks (20 days) showed significant improvements (except for anorexia) after receiving FMT, as presented in Table 4.

**Table 4:** Total score and scores of the six domains of the Irritable Bowel Syndrome-Symptom Questionnaire (IBS-SQ) in patients with IBS before and after fecal microbiota transplantation.

IBS-SQ	Screening	After FMT					P^a^	P^b^	P^c^	P^d^	P^e^	P^f^	P^g^
		Day 1	Day 2	Day 3	Week 1	Week 3							
**Total**	30.9±3.0	19.2±2.8	15.4±3	12.1±2.8	13±2.2	11.7±1.9	**<0.0001**	**0.0046**	**0.0003**	**<0.0001**	**<0.0001**	**<0.0001**	0.5
**Nausea**	3.5±0.8	2.8±0.8	1.3±0.5	1.9±0.6	2.2±0.7	1.3±0.5	0.07	0.4	**0.01**	0.08	**0.047**	**0.0013**	0.15
**Bloating**	7.9±0.5	4.9±1	4.7±0.8	3.1±0.8	3.5±0.9	3.2±0.8	**<0.0001**	**0.002**	**0.0002**	**<0.0001**	**<0.0001**	**<0.0001**	0.5
**Abdominal pain**	6.5±0.9	4.5±0.9	4.2±0.9	2.7±1	2.8±0.8	3.5±0.8	**0.0017**	**0.03**	**0.02**	**0.0009**	**0.0005**	**0.0005**	0.4
**Constipation**	4.2±1.0	2.6±0.8	2.6±1	1.4±0.8	1.5±0.6	1.4±0.6	**0.03**	0.3	0.07	**0.01**	**0.009**	**0.027**	0.8
**Diarrhea**	6.5±0.8	6.5±0.8	2.5±0.8	2.1±0.8	1±0.5	1.5±0.5	**0.0001**	**0.0018**	**0.0016**	**0.0004**	**<0.0001**	**<0.0001**	0.4
**Anorexia/ loss of appetite**	2.1±0.7	1.9±0.7	1.5±0.6	1.6±0.6	1.5±0.5	0.8±0.3	0.3	0.9	0.6	0.6	0.5	0.09	0.08

Data are presented as the mean±SEM. Comparison

^**a**^ one-way ANOVA with repeated measures; and Paired *t* test

^**b**^ Screening vs. day 1 post FMT.

^**c**^ Screening vs. day 2 post FMT

^**d**^ Screening vs. day 3 post FMT

^**e**^ Screening vs. week 1 after FMT

^**f**^ Screening vs. week 3 after FMT

^**g**^ Week 1 vs. Week 3.

IBS-SQ: IBS symptom questionnaire.

### Post-FMT complications

No complications were reported during and following FMT until the end of the study.

## Discussion

In this study of the kinetics of gut microbial community composition after FMT in IBS patients, the gut microbiota profile of the patients, which differed significantly from the donors before FMT, have shown some dynamic changes during the 28 weeks of follow up period post FMT. In addition, the patients complained of severe IBS symptoms and low QoL before FMT. The change in the gut microbiota in IBS patients during the course of the study parallels a rapid improvement in the patients’ symptoms and QoL that lasts up to 28 weeks.

The DI of the patients changed from severe dysbiosis before FMT to moderate dysbiosis after 12 weeks following FMT and maintained its new status throughout the course of the study. This showed that administrating the FMT via gastroscope in to the duodenum did not cause/worsen dysbiosis, on the contrary, it helped change the gut microbiota towards normobiosis. In general, dysbiosis in IBS is characterized by a decrease in *Actinobacteria*, *Bifidobacteria* and *Lactobacillus* [41], and an increase in *Bacteroidetes*, *Firmicutes* and *Proteobacteria* in the feces [41, 42]. Increased *Proteobacteria* in diarrhea-predominant IBS (IBS-D) including *E*. *coli* is associated with increased inflammation [41]. *Bifidobacteria* count is either decreased [41, 43] or increased in IBS patients [44]. In the current study, the bacterial signals for *Actinobacteria* in general and especially in *Bifidobacteria* in the total IBS group and the subgroups of IBS patients are significantly reduced compared to the donors’ group at the beginning of the study.

The gut microbiota profile in patients with IBS-D has a significant increase in bacteria belonging to the *Bacteroidetes* phylum and in *Ruminococcus* species [41, 45]. Increased *Ruminococcus* species may cause degradation of the mucus layer that allows infiltration of *Streptococcus* species causing low-grade inflammation [41]. In contrast to a previous study [24] that found no significant difference in the gut microbiota in IBS patients compared to donors other than a significant increase in *Streptococcus* counts in donors, we found significant differences in several bacterial signals in our IBS patients compared to their donors including a significant increase in bacterial signals for *Actinobacteria* and *Bifidobacteria* in the donors’ group. *Actinobacteria* and *Bifidobacteria* are important for gut mucosal barrier to keep pathogens from crossing over [41]. These alterations in the gut microbiota profile, especially that of *Ruminococcus gnavus*, *Proteobacteria* and *Shigella/Escherichia* might have contributed to the mechanism of low-grade inflammation in PI-IBS and IBS-D. *Proteobacteria* and *Shigella/Escherichia* signals in the recipients were significantly higher than that of the donors before FMT. *Proteobacteria* and *Shigella/Escherichia* signals further increased during the first week following FMT but then changed (decreased) towards values of the donors. No inflammatory changes were clinically noted among the recipients but one cannot exclude that a low-grade inflammation occurs in patients with IBS even from before FMT. The PCA scores show that FMT seems to have an effect on the gut microbiota as systematic change occurred from baseline before FMT and over a period of 28 weeks.

In a comparison of the gut microbiota profiles between the responders and non-responders groups, the changes in the bacterial signals of *Bacteroides*, *Desulfitispora* and *Megasphaera/Dialister* between the patients during the study are similar to or tend to change toward those measured for the donors at the beginning of the study. This may be explained by the fact that the donors and the patients are relatives and/or living in the same environment and also by the impact the donors’ gut microbiota may have on the patients’ gut microbiota. Another interesting observation is that the signals for *Bifidobacteria* in the responders group are lower than those in the non-responders group. Low *Bifidobacteria* signals have also been observed in IBS patients following a low-FODMAP diet as shown in a previous study [46].

The changes in the patients’ gut microbiota following FMT may have contributed to the subsequent improvement in their symptoms and thus QoL [25]. Similar observation has been recently described after using FMT to restore the bacterial diversity and resolve the dysbiosis in patients with recurrent *Clostridium difficile* infection [47]. Some of the bacterial strains changed towards the end of the study (weeks 12 and 20/28) without significantly worsening the symptoms of IBS and/or QoL during the same period. This may suggest that the changes in the symptoms and QoL may be related to the collective changes in the gut microbiota rather than individual bacterial change. The change in the gut microbiota profile of the patients at weeks 12 and 20/28 following FMT compared to the donors before FMT (Table 2) resembled that of the donors at the end of the study compared to the donors at the beginning of the study, which may have contributed to the increase in the DI towards the end of the study (Fig 2). The changes towards the end of the study are quite interesting as they raise a question to whether other factors may have influenced these changes such as the participants’ milieu, dietary (which were not changed according to the metadata) or hereditary factors; since the donors and the patients are relatives either living in the same environment (for example: spouse) or sharing the same genes (for example: a parent or a sibling) or both. Another explanation may have been due to the imposed changes to the patients’ gut microbiota following FMT rendering them susceptible to the same changes occurring to the donors’ gut microbiota.

FMT was associated in time with rapid improvement in IBS-SSS score (>50 points reduction from baseline) [29]. In the current study, 70% of the patients has improved IBS-SSS scores during the first week, 85% by 3 weeks, 70% by 12 weeks and 62% over 20 weeks towards the end of the study. The long lasting effect of FMT on IBS symptoms is in line with previous studies [23, 24]. In addition to an immediate improvement (during the first 3 days after FMT) in IBS-SQ–total and specific–symptom scores, namely; bloating, abdominal pain and diarrhea, a gradual but statistically significant improvement in all of the IBS symptoms as assessed by IBS-SQ (except for anorexia/loss of appetite) was observed on daily basis during the first 3 weeks following FMT. The improvements in IBS related QoL, abdominal pain and bloating during the course of the study is consistent with a previous report [24].

Currently, dietary manipulation is one of the methods for the management of IBS symptoms. [48–50] The response rate of an elimination diet ranges between 15 and 71% [51], and of low FODMAP diet is up to 86% [46, 48], with a high placebo response rate reaching to 40% [48]. One can suggest that FMT may serve as an alternative method for managing IBS instead of the dietary manipulation due to similar high response rate, easy to apply, and long lasting improvements in the symptoms and QoL up to one year [23, 24]. However, head to head comparison studies must be further conducted.

The route of administration of feces, either via gastroscopy [23] or colonoscopy [24] in to the upper or lower GI tract, respectively, have reported similar effects on IBS symptoms [23, 24]. In a systematic review of FMT in recurrent *Clostridium difficile* infection, the use of gastroscopy as an administration route had a response rate of 76% vs. 88% for colonoscopy [52]. In this study, we have chosen to use gastroscopy because it is an easy and a fast route of administration and because patients with IBS often have dilation of small bowel segments giving small intestinal bacterial overgrowth, SIBO, [53], which would have escaped the suspension had we chosen to use colonoscopy for FMT. The procedure is considered to be safe [31]. No complications were reported during and following FMT until the end of the study. In other studies, short-term adverse events occurred after FMT such as abdominal cramps, belching and nausea but they were self-limited and transient [23, 54]. Long-term, follow-up studies (3–68 months after FMT) have found FMT to be relatively free of adverse effects [55].

The strength of the study is the usage of validated methods to study the kinetics in the gut microbiota and validated questionnaires to assess the changes in the stool form, symptoms and QoL. The main limitations of the study are its design as an open-label trial, not placebo- or sham-controlled, and the small sample size. However, our main focus was to study the changes in the gut microbiota in the patients compared to the gut micobiota of the donors, which are supposed to have more-or-less stabile profiles during the study. Nevertheless, significant results were obtained despite the small sample size. Larger double-blinded, placebo-controlled studies are necessary to address applicability of FMT in IBS and are currently running else where in Norway (NCT02154867).

## Conclusions

This is the first study to investigate the kinetics of microbial community composition in IBS patients following FMT. FMT was associated with a rapid change in the alterations in the signals for several strains of the gut microbiota making it statistically not significantly different from the donors after 3 weeks following FMT. The gut microbiota profile at the end of the study was similar to the profile of the donors taken at the same time. The symptoms and QoL have improved significantly quite soon after FMT and lasted up to 28 weeks.

## Supporting information

S1 File FMT protocol-2013. (PDF)

S2 File Trend checklist. (PDF)
