# Supplementary material for: The kinetics of gut microbial community composition in patients with irritable bowel syndrome following fecal microbiota transplantation
Source: PLoS One. 2018 Nov 14;13(11):e0194904. doi: 10.1371/journal.pone.0194904 (PMC6235238; doi:10.1371/journal.pone.0194904)
Supplement: S1 File — (PDF) [file pone.0194904.s001.pdf]

FAECAL MICROBIOTA TRANSPLANTATION IN PATIENTS WITH IRRITABLE BOWEL SYNDROME

Our group and our network with contribution from each:
We enjoy the multidisciplinary contribution over years from the following groups, which will be involved in the present research initiative:

1. Prof. Trygve Hausken leader of the group for functional gastrointestinal disorders (FGID) at K1. He was also one of the leaders during the Giardia research project together with Prof Nina Langeland. He has a wide experience in IBS and particularly in post-infectious IBS. Together with prof Tom Hemming Karlsen he got Helse-Vest project grant in 2012,on the project "Gut microbiota in intestinal and systemic inflammatory conditions» and the present application is a part of this. Dr. Tarek Mazzawi is the main investigator in this project and is responsible for data analysis, statistics, planning and writing the manuscripts.
2. HUS Motility Lab led by Prof. Jan Hatlebakk, will provide the working area and equipment for motility, as well as post-processing analyses he is also a professor in nutrition and has his specialty in upper gastrointestinal disorders. He is responsible for planning and post-processing of data.
3. Prof. Odd Helge Gilja is leading a research consortium for visualization techniques in medicine, founded by the University of Bergen and Haukeland University Hospital. The National Centre for Ultrasound in GI Medicine (NSGU) and Medviz have been active in clinically oriented ultrasound research for many years. These groups are led by Prof. Odd-Helge Gilja and Prof. Trygve Hausken, and will contribute working facilities, recording equipment and post-processing of data.
4. Bergen Functional MRI Group, headed by Professors Kenneth Hugdal and Arvid Lundervold contributes equipment and expertise in fMRI recordings and analysis.
5. Prof. Magdy El-Salhy possesses world expertise in the neuroendocrine system of the gut, including the ghrelin axis and IBS.
6. Prof. Gülen Arslan Lied heads the food intolerance initiative in Bergen and has wide experience in elimination diets. She is responsible for planning and post-processing of data.
7. Prof. Tom Hemming Karlsen leader of the PSC group at the university of Oslo, leader of the project "Gut microbiota in intestinal and systemic inflammatory conditions». This project got funding from Helse-Vest 2012. He is responsible for the analysis of 16S rRNA sequencing of gut microbiota. He is also professor II at University of Bergen, K1.
8. Dr. Johannes Espolin Roksund member of the PSC group at the university of Oslo, and of the project "Gut microbiota in intestinal and systemic inflammatory conditions» responsible for the analysis of 16S rRNA sequencing of gut microbiota.

Background

Irritable bowel syndrome (IBS) is a common gastrointestinal disease, affecting 13-20 % of the adult population leading to significant morbidity and huge costs for the community (Hungin 2003). IBS is a long-lasting condition (>6 months), in most patients giving symptoms for several years. IBS is defined as abdominal pain or discomfort, which is associated with a change in bowel habits (Longstreth 2006). In the follow-up 5 years after the

Giardia outbreak in Bergen 2004, more than 50 % of the patients still had abdominal complaints (Hanevik 2009). Available therapies are limited but include psychological support, coping strategies and dietary interventions, and a few drug options are also available, depending on the individual symptom characteristics (diarrhea or constipation dominated IBS) (El-Salhy 2012). It is likely that a better understanding of the pathogenesis of the disease would open up for new treatment options.

The cause of IBS still remains unknown, and typical pathogenetic factors hypothesized to be involved are intestinal dysmotility, visceral hypersensitivity, immunological and psychosocial factors (El-Salhy 2012). Recent evidence suggests an important role of alterations in the gastrointestinal flora (Codling 2010, Tana 2010), and has led to an increasing interest in probiotic (Whorwell 2006, Sisson 2012) and antibiotic (Menees 2012) treatment approaches. The gut flora, at the taxonomic level denominated the gut microbiota (the bacterial gene content referred to as the gut microbiome), has recently been shown to be involved not only in gastrointestinal diseases, but also in a variety of systemic inflammatory and metabolic diseases (e.g. atherosclerosis and type II diabetes mellitus) (Ley 2006; Vrieze 2012, Karlsson 2012).

The gut microflora is a community that has co-evolved with the host and confers beneficial effects on human physiology and nutrition, and is crucial for human life {Backhed, 2005 #2036; Hooper, 2002 #2037}. Critical functions of the commensal flora include production of short-chain fatty acids, metabolism of nutrients and organic substrates {Scott, 2008 #2048}, the maintenance of intestinal epithelium {Falk, 1998 #2049}, protection against foreign pathogens and, importantly, the maturation and development of the immune system. Microbe–host relationships are tightly interrelated, such that host factors can induce functional changes in the microflora that, in return, affect host biology {Khor, 2011 #2051}. Dysregulation of normal co-evolved homeostatic relationships between gut bacteria and the host can therefore lead to altered metabolism and immune responses.

Fecal transplantation (FMT), the infusion of a fecal preparation from a healthy donor into the gastrointestinal tract of another human being is capable of altering the gut microbiome of the new host. There is speculation that human faeces from a healthy donor may constitute the ideal "probiotic", thus suggesting FMT as a treatment option for conditions where an altered microbiota has been detected, including IBS (Brandt 2013). For treatment resistant, antibiotic-associated *Clostridium* difficile colitis, FMT has been a treatment option at Haukeland University Hospital since 1998 and has consistently proven a safe and effective therapeutic option for these patients. No adverse effects were recorded. (Lund-Tønnesen 1998). In 2013 FMT is widely accepted as the recommended treatment for Cl.difficile enterocolitis not responding to standard antibiotic therapy (van Nood E, 2013). In the metabolic syndrome and in ulcerative colitis patients down to 7 years FMT has shown to be safe and effective. For other conditions, multiple trials are currently underway (e.g.
http://www.clinicaltrials.gov/ct2/show/NCT01650038 in ulcerative colitis,
http://www.clinicaltrials.gov/ct2/show/NCT01793831 in Crohn's disease,
http://www.clinicaltrials.gov/ct2/show/NCT01790711 in type II diabetes,
http://www.trialregister.nl/trialreg/admin/rctview.asp?TC=1776 in overweight, and studies are also being performed in children down to the age of 7 years.
The concept of FMT is not new in the medical literature. This idea was probably first used in veterinary medicine by the Italian anatomist Fabricius Aquapendente in the 17th century. However, there are reports of much earlier evidence of human FMT. During the Dong-Jin dynasty in the 4th century in China, Ge Hong, a well-known traditional Chinese medicine

doctor described the use of human fecal suspension by mouth for patients who had food poisoning or severe diarrhea. Despite the widespread application, little is known on the details of interactions between donor and recipient microbiota, the need for particular matching strategies, as well as the kinetics of induced changes as a function of these characteristics. Prior to the rational and ethically sound application of FMT in IBS, we consider the characterization of such pre-clinical parameters essential. In addition, there is a variety of microorganisms in stool, not only bacteria, and long-term consequences of stool installation therapy are therefore impossible to predict at present state of knowledge.

Approach:
On this basis, we propose to initiate a project first to characterize the biology of FMT in the context of IBS prior to wider clinical application of the method. Given the ethical concerns of any yet unknown adverse effects of FMT therapy, we will include patients only with moderate to severe symptoms. IBS is a heterogenous disorder and it is important to characterize the patients, and study as homogeneous patient populations as possible. We will therefore include post-infectious IBS patients after the Giardia outbreak and IBS patients with food hypersensitivity.

Objectives:
Primary objective:
To investigate the effect and safety of faecal microbiota transplantation in patients with irritable bowel syndrome.
Secondary aims: To investigate a detailed characterisation of donor and recipient microbial community composition (by means of 16S rRNA profiling) and determination of the kinetics of changes following FMT.

1. To investigate microbial kinetics following FMT, effect on symptoms and safety in an open pilot study. An open study is chosen in order to calculate number of patients needed in a planned controlled trial.
2. To study the effect of FMT on symptoms in a placebo-controlled trial. We will select patients with postinfectious-IBS after the Giardia outbreak in Bergen and patients with IBS with food hypesensitivity selected from the MAI group (Matallergi / intoleransegruppen).
In both groups we will use the patients´ own faeces as placebo. Characterisation of donor and recipient microbial community composition (by means of 16S rRNA profiling) will be investigated in both groups.

3. To study the effect of FMT treatment on epigenetics, quality of life, psychometric measures Biopsies will be taken before and after 12 weeks feces installation to study changes in molecular markers (RNA and immunhisteochemistry)

Project plan and Study design

Study 1 An open study, patients will receive FMT from a healthy family donor, and will be followed for 6 months with regard to safety parameters, as well as analysis of faecal samples.

Twelve patients who meet the Rome III criteria for IBS after the Giardia outbreak in Bergen 2004 with moderate to severe abdominal symptoms, will be included.

Study 2. To study the effect of FMT from healthy family members on symptoms in a placebo-controlled, multicenter trial. We will select patients with postinfectious-IBS after the Giardia outbreak in Bergen and patients with IBS with food hypesensitivity selected from the MAI group.
Number of patients needed from Study 2

Studie 3. To study the effect of FMT treatment on epigenetics, quality of life, psychometric measures Biopsies will be taken before and after 12 weeks feces installation to study changes in molecular markers (RNA and immunohistochemistry).

- **Exclusion criteria:**

History of inflammatory bowel diseases, gastrointestinal malignancy, blood in stool or antibiotic use within 1 month prior to FMT, immunocompromised patient defined as taking immuno-suppressive medications, history of opportunistic infections within 1 year prior to FMT, oral thrush, or disseminated lymphadenopathy. Patients who are scheduled for abdominal surgery, pregnant women or patients taking probiotics or taking antibiotics within 4 weeks prior to installation are also excluded from the protocol.

Inclusion criteria for donors:
Healthy, family member
Age > 18 years

Exclusion criteria for donors:
Pregnancy, use of pre/probiotics, history of inflammatory bowel diseases, IBS, chronic abdominal pain, or gastrointestinal malignancy, diarrhea, blood in stool or antibiotic and probiotic use within 1 month prior to FMT; immunocompromised defined as taking immuno-suppressive medications, history of opportunistic infections within 1 year prior to FMT, oral thrush, or disseminated lymphadenopathy.

Study procedures
Stool transplant donor screening: Donors of faecal flora are healthy individuals living in the same household as the patients. All donors are screened before donation for previous exposure to contagious infectious agents. Screening of blood included serologic testing for hepatitis A, B, C, HIV, EBV and CMV. All donor stool samples are cultured for enteric bacterial pathogens and screened for viruses and parasites.

Suspension of fresh faeces from a close family member will be instilled into the duodenum via an endoscope. We will use the per-oral route of installation because patients with IBS often have dilation of small bowel segments giving bacterial overgrowth. This route of administration has been safe in all studies (refs 1-3).
- Feces transplantation protocol: Fresh stool samples are obtained immediately before FMT. 60 ml of sterile 0.9% N-saline are added to a stool specimen with a weight of ~30 g and homogenized manually. A catheter is placed through a gastroscope into the lower part of

duodenum. 60 ml of faeces-suspension (screened from the patients view) are instilled via the catheter and thereafter flushed with 20 ml of sterile 0.9% N-saline.

- Stool samples will be collected twice before instillation (week - 4 and week 0), then after week 1, 4 and 12 and after 6 months. Fresh frozen dry stool is sampled at all time points, aliquoted and stored at -80 degrees (<24 hours from production to delivery).

- Microbial DNA analyses: Bacterial DNA will be extracted from stool using MoBio PowerSoil DNA extraction kit, and submitted to sequencing of the V1-V2 regions of the 16S rRNA gene with Illumina Miseq technology. Quality control and primary analysis of the sequences will be performed with the QIIME (qiime.org) open source software package. Measures of alpha and beta diversity (intra-individual and between group microbiol richness, respectively) as well as relative abundance of bacteria on different taxonomic levels (from phylum to genus level) will be calculated. Functional (metagenomic) content will be inferred from the microbiota profiles with the PICRUSt (http://picrust.github.io/picrust/) software.

- Blood samples (Hb, LPK, TPK diff, creatinine, ASAT, ALAT, INR and electrolytes) and urine sticks at each visit.

Symptom assessment:
Gastrointestinal symptoms and bowel habits will be recorded during a 12 week follow up period, at day 0, week 1, 4, 12 and week 28 for symptom assessment, patients are asked to report the severity of global IBS symptoms and each of the symptoms (diarrhoea, bloating, flatulence and pain) during the preceding week, using a four-point scale (0 = none, 1 = mild, 2 = moderate, 3 = severe). IBS Symptom Severity Scale (IBS-SSS) contains 5 questions that are rated on a 100-point visual analogue scale (VAS) (the severity of abdominal pain, the frequency of abdominal pain, the severity of abdominal distension, dissatisfaction with bowel habits, and interference with quality of life). The score ranging is from 0 to 500, with a higher score indicating the worse condition (scores < 175 represent mild IBS symptoms, 175 – 300 represents moderate severity, > 300 represent severe IBS). A decrease of 50 points on the IBS-SSS has been shown to correlate with improvement in clinical symptoms.

Psycometric measures (validated questionnaires):
SF-NDI (Nepean Dyspepsia Index)
HAD
EPQN

Efficacy assessment
Primary end point: Stool microbiota changes as measured by alpha diversity and abundance of major microbial taxa at 1, 4 and 12 weeks and 6 months after FMT

Secondary end points:
- Degree of long-term effect of fecal transplantation as measured by the proportion of patient stool microbiota originating from the donor at 1, 4 and 12 weeks after FMT
Global improvement in IBS symptoms at 4 and 12 weeks, and 6 months and as measured by IBS-SSS.
- Changes in frequency and consistency of stools (Bristol Stool Chart).
- Number of adverse events, as measured by a patient-reported adverse event form

Food Diary (prospective 4 days) at week 0 and week 4.

Statistical analyses:
The R package (http://cran.r-project.org) will be used for all analysis
For the primary end point, longitudinal analysis of microbiota composition will be performed with repeated measures ANOVA (for alpha diversity and bacterial abundances). If non-normal data, arcsine square root transformation or a nonparametric test will be used. Original donor and recipient stool microbiota will be compared (measures of alpha diversity and abundances of taxa) at baseline and at each recipient stool sampling time point using nonparametric or parametric methods.
For secondary end points, the proportion of patient stool originating from the donor will be estimated at each time point using SourceTracker (pmid 21765408). The donor feces will be investigated at baseline (alpha diversity, beta diversity, abundances of taxa) and at each time point after FMT.

Safety considerations:
We have long experience at Haukeland University Hospital in performing FMT for treatment-resistant *Cl. difficile* enterocolitis and have developed an extensive program for identifying safe donors of faeces for FMT. Over the years, no serious side effects have occurred (3). The subset of IBS patients who are eligible for the study are typically patients with a heavy symptom burden, with few treatment alternatives. We therefore consider the balance of safety concerns versus possibility of symptom improvement to be positive, and will stress the importance of collecting safety data on FMT before this treatment is becoming more widely used.

The patient will be monitored for 2 hours post infusion of faeces. Beyond this point adverse events will be patient reported at the time points of clinical follow-up.

Reported symptoms (n=number of patients)	Intensity of symptoms (mild, moderate, severe)	Over all	During FMT	During follow-up	Related to FMT
Bloating/Flatulence		n	n	n	
Abdominal cramping/ Pain/discomfort		n	n	n	
Diarrhoea		n	n	n	
Nausea/vomiting		n	n	n	
Fatigue		n	n	n	
Fever		n	n	n	

Compliance with strategic documents

The study program is within the area of unmet diagnostic and therapeutic needs in common disorders in the population. Helse Vest is currently proposing to establish a national competence service for functional gastrointestinal disorders at Haukeland University Hospital, and this will be a logical extension. This is in line with the stategic program for the Faculty of Medicine and Dentistry at the University of Bergen, which highlights its involvement in societal issues and cooperation with primary health care.

Relevance and benefit to society
These are prevalent conditions with loss in income, significant constraints on daily life activities and loss of productivity. The burden on the health care system is problematic. New diagnostic approaches and therapeutic targets are likely to reduce suffering and costs for patients and society alike. Research in IBS and FGID has often been industry-sponsored and has seldom addressed the underlying mechanisms. Except for studies originating from our group, research in this area in Norway has focussed on epidemiological and registry research.

Environment impact
None identified.

Ethical perspectives
These studies will aim to improve patient therapy and involve a combination of investigations that are considered safe and mostly non-invasive. The hazards involved as well as burden on each subject is considered acceptable. Loss of income and travel expenses will be reimbursed, as needed. Informed consent forms are being made and an ethics committee application is being filed. All data will be anonymized and full confidentiality will be respected according to rules and regulations, at all stages in data processing and the publication process.

Gender issues
We seek to recruit female researchers to our network to secure both equal opportunities and increased awareness of unmet health care needs in women.

Dissemination plan
Study results will be presented at national and international meetings including United European Gastroenterology Week and the international Neurogastroenterology and Motility meeting. Publications will be made in international peer-reviewed journals including *New England Journal of Medicine, Gastroenterology* and *Neurogastroenterology and Motility.* Results will also be presented at national and regional meetings for gastroenterologists and other health care providers, and for the national organisation for patients with GI disorders.

Functional GI disorders are of wide interest to the public, and for popular science reporting television, radio and health-oriented magazines with national distribution will be informed regularily.

There will be a website that is active throughout and after the study period, for study participants and professional and non-professional parties.

4.2 Communication with users

We run a patient school in FGID and courses for gastroenterologists and GI surgeons through which we will share our study results. The website will be important to communicate our findings as soon as they are released.

REFERENCES:

1. Hungin AP, Whorwell PJ, Tack J, et al. The prevalence, patterns and impact of irritable bowel syndrome: an international survey of 40.000 subjects. Aliment Pharmacol Ther 2003;17:643-50.
2. Longstreth GF, Thompson WG, Chey WD, et al. Functional bowel disorders. Gastroenterology 2006;130:1480-91.
3. Hanevik K, Dizdar V, Langeland N, Hausken T. Development of functional gastrointestinal disorder after Giardia lamblia infection. BMC Gastroenterol 2009;9:27.
4. El-Salhy M, Gundersen D, Hatlebakk JG, Hausken T. Irritable bowel syndrome. Diagnosis, pathogenesis and treatment options. Nova Science Publishers, Inc., New York, 2012.
5. Codling C, O'Mahony L, Shenahan F, Quigley EM, Marchesi JR. A molecular analysis of fecal and mucosal bacterial communities in irritable bowel syndrome. Dig Dis Sci 2010;55:392-7.
6. Tana C, Umesaki Y, Imaoka A, et al. Altered profiles of intestinal microbiota and organic acids may be the origin of symptoms in irritable bowel syndrome. Neurogastroenterol Motil 2010;22:512-19.
7. Whorwell PJ, Altringer L, Morel J, et al. Efficacy of an encapsulated probiotic Bifidobacterium infantis 35624 in women with irritable bowel syndrome. Am J Gastroenterol 2006;101:1581-90.
8. Sisson G, Ayis S, Bjarnason I. Treatment of irritable bowel syndrome with a multi-strain probiotic: a randomized, double-blind, placebo controlled trial. Gut 2012;61(Suppl 3):A103
9. Menees SB, Maneerattannaporn M, Kim HM, Chey WD. The efficacy and safety of rifaximin for the irritable bowel syndrome: a systematic review and meta-analysis. Am J Gastroenterol 2012;107:28-35.
10. Ley RE, Turnbaugh PJ, Klein S, Gordon JI. Microbial ecology: human gut microbes associated with obesity. Nature 2006;444:1022-3.
11. Vrieze A, van Nood E, Holleman F, et al. Transfer of intestinal microbiota from lean donors increases insulin sensitivity in individuals with metabolic syndrome. Gastroenterology 2012;143:913-6.e7.
12. Karlsson FH, Fåk F, Nookaew I, et al. Symptomatic atherosclerosis is associated with an altered gut metagenome. Nat Commun 2012;3:1245
13. Backhed F, Ley RE, Sonnenberg JL, et al. Host-bacterial mutualism in the human intestines. Science 2005
14. Hooper LV, Littman DR, Macpherson AJ. Interactions between the microbiota and the immune system. Science 2012;336:1268-73.
15. Falk PG, Hooper LV, Midttvedt T, et al. Creating and maintaining the gastrointestinal ecosystem: What we know and what we need to know of the gnotobiology. Microbiol Mol Biol Rev 1998;62;1157-70
16. Khor C-C, Hibberd ML. Revealing the molecular signatures of host-pathogen interactions. Genome Biol 2011:12:229
17. Brandt LJ. American Journal of Gastroenterology lecture: Intestinal microbiota and the role of fecal microbiota transplant (FMT) in treatment of C.difficile infection. Am J Gastroenterol 2013:108:177-85.
18. Kunde S, Pham A, Bonczyk S, et al. Safety, tolerability, and clinical response after fecal transplantation in children and young adults with ulcerative colitis. J Ped Gastroenterol Nutr 2013. DOI: 10.1097/MPG.0b013e318292fa0d.
19. Lund-Tønnesen S, Berstad A, Schreiner A, Midtvedt T. Clostridium difficile-assosiert diaré behandlet med homolog feces. Tidskr Nor Laegeforen 1998;118:1027-30.
20. van Nood E, Vrieze A, Nieuwdorp M, Fuentes S, Zoetendal EG, de Vos WM, et al. Duodenal infusion of donor feces for recurrent Clostridium difficile. The New England journal of medicine. 2013;368(5):407-15. Epub 2013/01/18. doi: 10.1056/NEJMoa1205037. PubMed PMID: 23323867.
